# Geographic Market Definition in the Merger Guidelines: A Retrospective Analysis

**DOI:** 10.1007/s11151-018-9658-4

**Published:** 2018-09-15

**Authors:** Kenneth G. Elzinga, Vandy M. Howell

**Affiliations:** 1 University of Virginia, Charlottesville, USA; 2 Cornerstone Research, San Francisco, USA

**Keywords:** Antitrust law, Horizontal mergers, Market structure

## Abstract

Since the initial Merger Guidelines in 1968, the Department of Justice and Federal Trade Commission have revised their merger enforcement screen over the course of six versions. This article examines the evolution of the geographic market component of the Guidelines and the economic implications of changing standards of market delineation on merger enforcement. Using an illustration from the beer industry, we chronicle the development of geographic market definition and its varying effects on merger enforcement over the past 50 years.

## Introduction

The enforcement trajectory of the six Horizontal Merger Guidelines—1968, 1982, 1984, 1992, 1997, and 2010—serves as a chronicle of how merger enforcement has changed over the last 50 years. To simplify the evolution somewhat: In the 1968 Guidelines, mergers were prohibited if they increased concentration (even a little), and the taproot concern was that increased concentration would lead to joint action between firms that would harm consumers: collusion, whether tacit or direct, between the merging firm and other sellers in the relevant market. By the time of the 2010 Guidelines, it was well understood that mergers can benefit consumers; and while increases in concentration remained a concern, more attention was paid to unilateral conduct: whether a merger would enable the exercise of market power by the merged firm’s acting unilaterally.

As the Guidelines evolved, they introduced the hypothetical monopolist test; afforded more consideration to the cost-savings that a merger might generate; and clarified how entry conditions matter when evaluating a merger. While the Guidelines today have greater specificity than when they were first issued, their framework still enables the enforcement agencies to exercise discretion in their exegesis.

A remarkable feature of the Guidelines is the fact that they have changed so substantively over time, while the text of Sect. 7 of the Clayton Act has remained the same. Though they are not law *de jure*, the Guidelines have come to be law de facto. The Guidelines receive attention and use outside of agency merger approval and litigation. The language of the Guidelines is of consequence beyond its adoption and use by antitrust authorities and is regularly cited and used to shape analyses in contexts that are unrelated to the scrutiny of mergers and acquisitions. The Guidelines’ paradigm influences how a variety of practitioners and decision makers address questions of competition and monopoly.

To make sense of contemporary horizontal merger enforcement—and other topics in antitrust enforcement as well—it is fruitful to look back and see how we arrived where we are. In particular, in this paper we review the six editions of the Guidelines and describe the evolution over time of how relevant geographic markets have been defined—as well as the role of geographic market definition in assessing the prospect of merging firms exercising market power.^1^ To illustrate the changes we also offer an example that explains some ways in which the various versions of the Guidelines have differed in their stated approach to geographic markets. Using the Pabst Brewing Company and Blatz Brewing Company merger that was challenged by the Department of Justice (DOJ) in 1959, we show what facts might have been expected to be emphasized differently over time were one to follow the changes in the language of the Guidelines.

Conceptually, the definition of the relevant geographic market is as important as the definition of the relevant product market. To assess whether a merger is likely to cause harm or bring benefits to consumers, or to consider whether a firm or group of firms has geographic market power in general, one must know what market forces currently, or in the future, would thwart any attempt to increase prices (either unilateral or collusive). The answer to this question depends, among other things, on identifying the set of existing and potentially entering firms that would compete with the merging firms (or firms in question). Geographic market definition is the use of economic analysis to identify that set of firms. Which of the competitors that can or do sell the relevant products at issue could or will constrain pricing? Only the one down the street? All firms in the county, or the state, or the country? Getting the answer to these questions right is just as important as getting product market definition right.

Over the history of the Guidelines, questions about geographic market definition have been secondary to questions about product market definition. This is reflected in the various versions of the Guidelines, where the methods for identifying the geographic scope of competition are always discussed second, and have sometimes been described in the Guidelines as an exercise that requires using identical methodologies to those used in identifying product markets—without emphasis on the specific details that may require attention when defining geographic markets.

In particular, the analysis of geographic markets can be especially important in markets for in-person services, where demand or supply may not as easily shift across geographic space in response to price changes.^2^ Many products can be readily moved about in geographic space. People sometimes cannot. In service markets such as healthcare, the analysis of the relevant geographic market may be more important than the relevant product market.

In describing the evolution of geographic market definition over the history of the Guidelines, one cannot totally set aside how the Guidelines define the delineation of relevant product markets and their participants. There are two reasons for this: (1) Product markets and geographic markets are, as a matter of theory, defined conjointly; and (2) Identifying the set of market participants is critical for defining the relevant geographic markets (and vice versa).^3^ Nevertheless, this paper’s focus is that of how geographic market definition is spelled out in the Guidelines over time.

## The 1968 Guidelines

The first case that was brought by the DOJ under the amended Sect. 7 of the Clayton Act to the Supreme Court was *Brown Shoe Co. v. U.S.* (1962). The Court upheld the DOJ’s challenge to this merger based upon a “desire to promote competition through the protection of viable, small, and locally owned business.”^4^ While the Court conceded, “congressional concern [was] with the protection of *competition*, not *competitors*, and its desire to restrain mergers only to the extent that such combinations may tend to lessen competition,” protecting competition at the time meant pursuing the protection of the number of competitors.^5^ The 1968 Guidelines were in the grain of *Brown Shoe.* While *Brown Shoe* has not been overturned by the Court, later versions of the Guidelines essentially have ‘overturned’ the 1968 version with regard to how markets should be defined and how market structure is to be interpreted.

The stated purpose of the 1968 Guidelines was to “acquaint the business community, the legal community, and other interested groups and individuals with the standards currently being applied by the Department of Justice in determining whether to challenge corporate acquisitions and mergers under Sect. 7 of the Clayton Act” (DOJ 1968a, b, p. 1). Because the Guidelines based enforcement policy (i.e., which mergers the DOJ would challenge) on market structure, the definition of the market became the key to merger enforcement. Specifically, the Guidelines began by devoting an entire section to market definition, before discussing other aspects of merger analysis.

The DOJ defined markets ambiguously in the 1968 Guidelines, stating that a market was “any grouping of sales (or other commercial transactions) in which each of the firms whose sales are included enjoys some advantage in competing with those firms whose sales are not included” (DOJ 1968a, b, pp. 2–3). At this point in time, a relevant market was to be “defined both in terms of its product dimension (‘line of commerce’) and its geographic dimension (‘section of country’),” without establishing any analytical connection between these two elements of merger review (DOJ 1968a, b, p. 3).

The 1968 Guidelines defined geographic markets ambiguously as well, calling them an area (or areas) in which “firms engaged in selling the product make significant sales of the product to purchasers in that section or sections” (DOJ 1968a, b, p. 4). The initial version of the Guidelines offered no specific guidance as to how to determine a threshold of “significant sales” that would establish the firms to be included in the relevant market, or when several markets shall be defined, beyond the presence of “data limitations or other intrinsic difficulties” (DOJ 1968a, b, p. 4).

The 1968 Guidelines defined a product market as “the sales of any product or service which is distinguishable as a matter of commercial practice from other products or services,” and asserted that several different products may be included in a single market—provided that they are reasonably interchangeable.^6^ As with geographic market definition, product market definition was vague as to the factual patterns that would delineate a market; the Guidelines only highlighted that product interchangeability should be evaluated in terms of “price, quality, and use.”^7^

The 1968 Guidelines established consumer substitution patterns as central to product market definition. In theory, it could be considered an identical exercise to identify which products, and which geographic areas, are ‘interchangeable’ in a buyer’s eyes. But the 1968 Guidelines offered little specific guidance as to the kinds of data that were to be considered relevant in drawing the boundaries of either product or geographic competition.

Economic analysis teaches that transportation costs, relative to the value of the product, and the absence of legal barriers (such as regulations, tariffs, and quotas) are especially relevant variables in delineating the boundaries of geographic markets. The relative cost of transportation can be assessed not only with regard to shipping the product or service but also to the relative cost of transporting the buyer or consumer. Factors such as these were not explicitly delineated until the 1982 Guidelines as a list of relevant variables that the DOJ would consider when evaluating product and geographic substitutability (DOJ 1968a, b, p. 5, 9). These lists clarified the different scope of geographic and product market definitions, and also were used to address changes in the methodology of geographic definition. Overall, while the 1968 Guidelines made clear the importance of geographic market definition, they lacked any specificity in the implementation defining geographic markets.

Some observers today view the 1968 Guidelines as a reflection of the prevailing “populist emphasis on preserving fragmentation, local control, small business, etc.,” given that the thresholds of concentration that invite scrutiny were exceptionally low as compared to now, and it is highly unlikely that any antitrust economist would urge their adoption today (Skitol and Vorasi 2012, p. 49). However, many of the precepts of the initial version were reflections of what the Court wrote about merger policy in *Brown Shoe*. At that point in time, there was not a wedge between the Court and the DOJ with respect to merger enforcement. This affinity was not, however, a stable condition.

## The 1982 Guidelines

Of all the revisions of the Guidelines, there was no greater change than from 1968 to 1982. Under the direction of Assistant Attorney General William Baxter, the 1982 Guidelines reflected changes in economic scholarship. The 1982 Guidelines exhibited more interest in the economic reasons to tolerate mergers that increase market concentration as well as recognition of the importance of the prospect of generating production efficiencies. The thematic change is reflected in the opening statement which is congenial towards mergers, not hostile to them: “Although they sometimes harm competition, mergers generally play an important role in a free enterprise economy”. In other words, instead of mergers’ generally being presumed to be anticompetitive no matter the market structure, the 1982 Guidelines suggested that one might now generally presume that mergers led to efficiencies—unless it was shown to be otherwise.^8^

The 1982 Guidelines introduced several changes from the 1968 version: The most important conceptual change was the Hypothetical Monopolist Test (HMT) for market definition; and the most important quantitative change was the Herfindahl–Hirschman Index (HHI) for measuring market concentration after a relevant market was defined. The 1982 Guidelines also included new discussions that identified market participants and outlined how firms might engage in post-merger coordinated conduct.

In the simplest sense, the 1982 Guidelines softened the presumption about what market conditions might be expected to be harmful to consumers, but they also increased the specificity as to what kind of analysis should be conducted to assess whether these market conditions existed. The new Guidelines proposed following a logical process: One should first apply a specific analysis rooted in economic theory to define the scope of the product and geographic market in which the merged entity competes, and then assess specific empirical criteria within that market to assess the merger’s potential impact. Whatever text could be found in the 1968 Guidelines that based merger analysis on protecting competitors was not to be found in the 1982 version; the new version was all about protecting and promoting competition itself.

The 1982 Guidelines first set forth the hypothetical monopolist paradigm that was hinted at in the Court’s 1956 *Cellophane* decision and offered this as the chosen test to define both product and geographic markets (Werden 2003).^9^ Werden (2003, p. 254) contends that the first original recognition of this test was put forward by Morris Adelman in a 1959 law review article.^10^ Specifically, the 1982 version defined a market as “a group of products and an associated geographic area such that (in the absence of a new entry) a hypothetical, unregulated firm that made all the sales of those products in that area could increase its profits through a small but significant and non-transitory increase in price (above prevailing or likely future levels)”. This language makes clear the conjoint nature of product and geographic market delineation. The 1982 Guidelines also defined a hypothetical price increase of 5% as the “small but significant and non-transitory increase in price” (SSNIP) in order to assess and delineate both product and geographic markets.

Antitrust lore has it that the 5% figure did not come from economists at the DOJ but rather from William Baxter himself. Even if untrue, the story is worth telling: When asked how large the price increase should be in a SSNIP test, Baxter looked at his hand, considered the number of fingers, and said, “five percent.” Whatever is the taproot of the number itself, considered as a *Gedanken* experiment or applied by statistical inference, the SSNIP test became the analytical peg on which the 1982 Guidelines and all subsequent versions would hang.

Under the 1982 Guidelines, geographic markets, like product markets, were defined as areas such that “a hypothetical firm that was the only present or future producer of the relevant product in that area could profitably raise price” by at least 5%. But *where* does one start when applying this test with regard to geography? This question would continue, through all the versions of future Guidelines, to be of essential importance. In effect, the 1982 version asserted that a regulator would first identify a “provisional geographic market based upon the shipment patterns [of the] firm and its closest competitors,” reflecting the use of the Elzinga–Hogarty test at the time.^11^ Because such a provisional market would be based on pre-merger prices, it might be expected to exclude additional firms that would sell the relevant product post-merger were there to be an increase in price. In such a situation, the 1982 Guidelines stated that the DOJ would “if necessary, expand the provisional market” to include the locations of firms that *could* supply to customers that would switch following a SSNIP.

The 1982 Guidelines directly spelled out the list of relevant evidence to consider when evaluating whether to expand the provisional geographic market, which included:

- Buyers who actually shifted their purchases among sellers at different geographic locations, especially if the shifts resulted from changes in relative prices;
- Similarities or differences in the price movements of the relative product in different geographic areas over a period of years;
- Transportation costs;
- Costs of local distribution;
- Excess capacity by firms outside the provisional market.

The 1982 Guidelines also mentioned that the DOJ would consider the existence of narrow geographic markets where geographic price discrimination was practiced (or would likely be practiced) against particular buyers as a result of the merger. The 1982 Guidelines mentioned as an example a firm that “charg[es] different prices net of transportation costs for the same product to buyers in different locations,” but did not develop or explore this idea in empirical detail. This new feature foreshadowed the later Guidelines’ increased emphasis on the concept of price discrimination generally.^12^

Having adopted a new methodology of market definition and the identification of relevant participants within those markets, the 1982 Guidelines then adopted more sophisticated measures of market concentration for the purpose of enforcement policy. In a nutshell, the operative economic principle was that certain levels of concentration are more likely to lead to collective behavior that harms competition and those mergers that result in those levels of market concentration would likely be challenged. Rather than the four-firm concentration ratio, the HHI became the preferred measure of market structure, classifying markets as “unconcentrated,” “moderately concentrated,” and “highly concentrated,” and establishing HHI thresholds for merger challenges in each silo of market concentration.^13^ ^14^ ^15^ Of course, calculating an HHI is worth the candle only if the product and geographic contours of the relevant market make economic sense.

Under the 1982 Guidelines’ ‘Leading Firm Proviso,’ the DOJ also indicated that the agency would challenge mergers that did not meet the HHI thresholds in instances when the transaction involved a merger between a firm with at least 35% market share and any firm of at least 1% market share. A ‘leading firm’ could fall well short of being a dominant firm and still be considered ‘leading.’^16^

While the 1982 Guidelines offered notable changes to the measurement and thresholds of market concentration, the Guidelines cemented the centrality of market definition because market shares cannot be determined without first defining a relevant market. With regard to geographic market definition, on the one hand, the 1982 Guidelines took strides to institute an analytical framework based upon the HMT and provided clear specification of which facts were relevant evidence for the definition of geographic markets.^17^ On the other hand, the 1982 Guidelines still excluded discussion of some of the key facts that are involved in showing the existence of price discrimination, which is an often critical step to determining the existence of narrow geographic markets.

## Guidelines

The difference between the 1968 and 1982 Guidelines was substantial. In comparison, the changes from 1982 to 1984 were a mopping up exercise—though they did affect the task of geographic market definition. Specifically, the 1984 version explicitly introduced an iterative algorithm as the methodology that would be used to implement the HMT in order to define product and geographic markets, and allowed for greater flexibility in applying the SSNIP. The 1984 Guidelines preserved the identification of market participants set forth in the 1982 version.

While use of the HMT for market definition remained in the 1984 Guidelines, implementation of the HMT was altered to incorporate the “next-best substitute” principle. The 1982 Guidelines did not prescribe the order in which different products or geographic territories were to be considered as candidates for the HMT. In contrast, the 1984 Guidelines, when assessing candidate markets, began with the merging firm’s product, and iteratively tested the ‘next-best’ substitute product.

When defining geographic markets, this meant a departure from using the 1982 approach of measuring physical shipments to identify first a provisional geographic market (DOJ 1984, p. 9). Rather, the 1984 Guidelines stated that the focus should be on the location of the merging firms’ facilities as a starting place.^18^

To illustrate: Imagine two potential merging firms that sold the same product (e.g., widgets) nationally, but were both located in Maine. In this example, the HMT would now begin in Maine and iteratively test through the SSNIP the ability of other *existing* firms in other locations to serve the national customers of those Maine firms. Importantly, which of these firms’ customers—e.g., all customers in all locations? only customers in the state?—should be included in this geographic market exercise was not made explicit in the Guidelines.^19^ In this example we use all customers of the Maine firms. If the first iteration of the SSNIP did not determine the boundaries of a Maine geographic market (in our example, imagine a firm in Vermont could defeat a SSNIP from the two Maine firms by selling to the Maine firms’ national customers), the analysis would assume a larger geographic market that included Vermont and move to the next location “from which production is the next-best substitute” for consumers. It would do so until the “smallest market” that could sustain a hypothetical monopolist’s price increases was identified (DOJ 1984, p. 9).

Two immediate issues arose: First, the 1984 Guidelines did not provide a methodology for determining what the next-best geographic substitute for production was. The algorithmic approach was implemented with the intent to ensure that product and geographic markets could not be constructed to include or test more distant production facilities or less substitutable products first while omitting closer substitutes. In our example, this might happen if one tested whether a firm in Hawaii could discipline the two potential merging Maine firms—before testing whether a firm in Vermont could do so. This was done with the intent of preventing an overstatement of the market share of potentially merging firms.

Second, the strict focus on existing production locations for defining geographic candidate markets had limited corresponding detail with regard to implementation (e.g., which customers are included in each iteration, at what unit of geography to begin). This was coupled with a lack of explicit connection to the list of relevant evidence that is contained in these Guidelines, which indicated that the shipment patterns (e.g., the locations of customers) provide evidence for geographic markets. Given the widespread use of the Guidelines in various types of antitrust litigation, the lack of specificity in the text allowed for different interpretations on how a geographic market ought to be defined.

Consider the following illustration in the context of Guidelines’ language: The 1982 Guidelines’ approach, which continues to exist in the 2010 Guidelines, essentially considers production from a *new location* (e.g., outside the geographic market as defined by the location of existing producers) as equivalent to competition from a *new product* (e.g., outside the product market as defined by existing products). These may be parallel in a strict theoretical sense; but as a matter of economic intuition, they are not. A literal application of the HMT algorithmic approach that is in the Guidelines indicates that the location of production is what determines the scope of the geographic market. But what does a producer-location geographic market *mean* without including or considering the geography of its customers? To ignore customer location is to ignore important information about market contours.

Imagine (based on our example above) that the only existing producers of widgets were in Maine and Vermont, but their customers were located throughout the United States. If there were no other existing suppliers outside of Maine and Vermont, the text of the Guidelines would suggest that the analysis could and/or should stop there, and thus could define a “geographic market” of an area ‘including Maine and Vermont’ (and states in between), but not the nation. Yet isn’t the ‘proof in the pudding’ already that the geographic scope of competition is national for widgets? Unlike the need to assess whether customers would be interested in a new product, hasn’t the nationwide location of existing customers of widgets already answered that question with regard to geography? Couldn’t one simply assume that customers being served nationally by the existing Maine and Vermont firms would welcome widgets from other locations as well and that these also would be ‘in the relevant market’?^20^

These issues around the scope of geographic competition can be considered conceptually different than the more difficult question of determining whether a new, not-yet-existent product would compete in a given product market. The lack of intuitive appeal to the ‘production location only’ approach to defining geographic markets in the text of the Guidelines can lead to considerable variation in how practitioners approach defining geographic markets in the context of litigation.

The 1984 Guidelines also increased flexibility in the application of the SSNIP test. While in most merger investigations, the DOJ would continue to use a price increase of 5% that lasts for 1 year (as in 1982), the DOJ could now use a price increase that was larger or smaller, depending on the nature of the industry (DOJ 1984, p. 5).

Some commentators praised the 1984 Guidelines for shifting the focus of market definition towards the measurement of potential anticompetitive effects of mergers by adding further specificity to the market definition methodologies when compared to the 1982 Guidelines (Scheffman and Spiller 1987, p. 125). However, some commentators argued that the rigid, iterative approach to implementing the HMT that was specified in the 1984 Guidelines could be inconsistent with business reality and hard to implement in practice (Shapiro 2010, p. 88). For the task of geographic market definition, the 1984 Guidelines dramatically changed the starting point for a candidate market, while continuing to offer a distinct discussion of some of the types of evidence that are especially important when considering an expansion of the boundaries of a geographic market.

## The 1992 Guidelines

As the Merger Guidelines became increasingly prominent in antitrust enforcement, the Federal Trade Commission (FTC) joined as an author. The 1992 version of the Guidelines was issued jointly by the DOJ and the FTC. This made transparent what already was happening behind the scenes: Economists at both the Federal Trade Commission and the Antitrust Division were relying on the Guidelines’ paradigm in their merger analyses. But the 1992 Guidelines were important for other reasons than making public the rapport between the FTC and the DOJ. The Guidelines made important changes with regard to focusing on unilateral effects, but also took a step backward in terms of clarity around geographic market definition.

While earlier versions of the Guidelines focused on coordinated effects, the 1992 version devoted important time and attention to unilateral effects: the effect of a merger on market price or output that might occur even in the absence of post-merger coordination between the merging firm and other sellers in the market.^21^ The emphasis on unilateral effects was provoked by concern at the enforcement agencies that many mergers were in markets with differentiated products—where the elimination of a rival (though acquisition) that was close in product space could enable the acquiring firm to exercise market power unilaterally. The concern with unilateral effects was driven by economic theory: particularly models that are based on a Bertrand oligopoly in markets with differentiated products (Shapiro 2010, p. 54). In pursuing this aspect of merger analysis, geography was subordinated to product definition—even though the Bertrand model, in theory, can be equally applied to spatial differences. But geography was not explicitly discussed in this version of the Guidelines.^22^

The introduction of unilateral effects in the 1992 Guidelines changed the Agencies’ merger enforcement policy: Specifically, in markets where unilateral effects were likely to lessen competition and increase prices (i.e., markets where merging firms produced differentiated products or where merging firms produced a homogenous product but were differentiated in productive capacity), the 1992 Guidelines indicated that even if the merging firms’ apparent market shares remained outside the enforcement thresholds, the merger might still be challenged.^23^ The discussion of potential unilateral effects due to differentiation did not mention specifically how differences across geography might interact with changes to market power as a result of a merger.

While the recognition of unilateral effects offered a new focus for merger policy in the 1992 Guidelines, market definition remained central to merger enforcement, as did the use of the HMT for defining geographic markets. Discussion of market definition was the first section of the 1992 Guidelines, and an assessment of “whether the merger would significantly increase concentration and result in a concentrated market, properly defined and measured” remained the first step in the Agencies’ analytical process for merger review (DOJ and FTC 1992, p. 3).

Between the 1984 and 1992 Guidelines not only did market definition remain center stage, but there also were important changes in the recommended steps to defining a market. The 1992 version asserted that market definition “focuses solely on demand substitution factors,” (DOJ and FTC 1992, pp. 3–4) whereas the 1984 Guidelines “evaluate both the probable demand responses of consumers and the probable supply responses of other firms” (DOJ 1984, p. 3). The 1992 Guidelines took the approach that one could define the market without assessing supply responses. Alfred Marshall presumably would have found this puzzling. But if Marshall were to read the 1992 Guidelines closely, he would find that they did not entirely ignore the supply side of a market. Instead, supply responses would be taken into account when identifying the market’s participants—a process that is separate from the initial market definition.

Most of the key changes in the 1992 Guidelines regarding market definition flowed from the decision to focus only on demand substitution factors. First, the 1992 Guidelines clarified the “next best substitute” principle of the HMT that was introduced in the 1984 Guidelines. The 1992 version explicitly indicated that the market for which the “greatest value of diversion of demand” is expected following an increase in price should be tested as the next candidate market when iteratively defining product and geographic markets (DOJ and FTC 1992, p. 6).^24^

Second, and related, the 1992 Guidelines changed how the Agencies identified market participants, with greater emphasis placed on potential supply responses to the merger: e.g., supply responses that were no longer considered when defining the market itself. The 1992 Guidelines proposed a more detailed analysis of the entry of firms into the relevant market post-merger: The Guidelines define entry as “easy if entry would be timely, likely, and sufficient in its magnitude, character and scope to deter or counteract the competitive effects of concern” as potential deterrents of anticompetitive effects of a merger (DOJ and FTC 1992, p. 24). Relevant entrants were ones that could, with minimal sunk cost, enter the market and begin production of the product within a year (DOJ and FTC 1992, p. 5).

The choice to remove supply side responses from the geographic market *definition* exercise, combined with a producer-focused algorithmic SSNIP, led to some tough definitional questions. Let us return to our example of a Maine and Vermont producer-based geographic market: What if, in that hypothetical, in response to the merger of the two Maine firms, there was *expected* to be a potential *new* supplier in Georgia that would enter and discipline a SSNIP of the Maine and Vermont firms to their national customers? How would one describe this dynamic? The 1992 Guidelines, taken literally, would argue that the expected future supply response from this Georgian firm is not relevant to geographic market definition. The entering Georgian firm could only be a market participant that would be considered *after* the geographic market was defined. In other words, this geographic market would be defined as having future supply-side participants who were not physically located within the boundaries of the geographic market itself.

Antitrust practitioners and decision makers could be forgiven if they found this confusing. What would it mean to have a geographic market limited to Maine-Vermont but that could be disciplined by potential new entrants outside it? How helpful is it to a decision maker to consider a geographic market constructed in this manner?^25^ In reality, the lack of intuitive economic logic in the 1992 Guidelines in regard to geographic market definition led practitioners to take a variety of different HMT approaches to defend different “geographic markets” in antitrust contexts, even if the Agencies had a specific interpretation in mind.

The 1992 Guidelines also ceased to treat geographic market definition as something distinct from product market definition altogether, no longer mentioning or requiring specific analysis that is targeted at geography. Removed as important to the task of defining geographic markets was earlier Guidelines’ text such as “the shipment patterns of the merging firm and those firms with which it actually competes for sales… transportation costs, costs of local distribution, and excess capacity of firms outside the location of the merging firms” (DOJ 1984, p. 10). Instead, the new Guidelines replaced the separate list of evidence considered pertinent for defining relevant product and geographic markets in the 1984 Guidelines with the following list of relevant evidence for *both* product and geographic markets^26^:

- Evidence that buyers have shifted or have considered shifting purchases between products/geographic locations in response to relative changes in price or other competitive variables;
- Evidence that sellers base business decisions on the prospect of buyer substitution between products/geographic locations in response to relative changes in price or other competitive variables;
- The influence of downstream competition faced by buyers in their output markets; and
- The timing and costs of switching products/suppliers.

The shift in the 1992 Guidelines to assessing only demand substitution factors when defining relevant geographic markets had limitations. Even though the Guidelines accounted for entry conditions when assessing a merger’s anticompetitive effects and delineating market participants, the geographic market definition itself was not adjusted to account for entrants. As shown above, this could generate a peculiar if not anomalous geographic market definition and, as a consequence, an ill-advised merger enforcement decision.

Proper geographic and product market definition requires understanding how demand *and* supply would respond to potential price increases that stem from anticompetitive activity—not just a demand-side response. This is a basic principle of economics, going back to Alfred Marshall’s *Principles of Economics,* where he put forward his scissors analogy to explain that both demand and supply were necessary to the process of price determination (Marshall 1961, p. 348).

## The 1997 Guidelines

The 1997 Guidelines included a

“substantial revision and expansion … of the treatment of merger efficiencies … [reflecting] an appreciation that mergers can promote competition by enabling efficiencies, and that such efficiencies can be great enough to reduce or reverse adverse competitive effects that might arise in their absence” (Shapiro 2010, pp. 54-55).

With the structure and language of the document nearly identical to the 1992 Guidelines, the 1997 version contained no changes as to enforcement policy, no changes as to the definition of product or geographic markets, and no changes as to the identification of competitor firms. Geographic market definition continued to be a central exercise to assess a proposed merger, although it was now treated essentially as a theoretical exercise that is no different than that of defining a relevant product market. The 1997 Guidelines maintained the ambiguity present in the 1992 Guidelines as to geographic market definition.

## The 2010 Guidelines

It takes a little unpacking to understand the impact of the 2010 Guidelines on the exercise of geographic market definition. While the 2010 Guidelines offer a variety of changes and clarifications about how to define a geographic market, they also mark a departure from the historic focus on first defining a market, and then assessing changes in concentration. The 2010 Guidelines emphasize that “merger analysis does not consist of uniform application of a single methodology.” At one level, nothing new is being said here. From the beginning, the DOJ, and then the FTC, make clear that government enforcement is not tied to the Guidelines in some wooden and mechanical manner. But at another level, the 2010 Guidelines represent a paradigm shift: The analytical center stage is now devoted to attempting to measure directly the economic consequences of horizontal mergers rather than inferring the consequences from implied changes in market structure.^27^ To the extent that this was a protocol already being followed within the enforcement agencies, the protocol is now explicit and public.

The 2010 Guidelines incorporate analytical tools that the Agencies will use to assess competitive effects, indicate an openness to more diverse forms of evidence, and remove the algorithmic description of the HMT. These changes move the Guidelines’ focus away from a one-size-fits-all analytic approach and towards an acknowledgement of the appropriateness of using a variety of methods to measure the price changes that consumers may face as a result of a merger. In this shift, both product and geographic market definition, previously considered the first steps of any merger analysis, are now viewed as secondary to an understanding of the competitive effects of a merger. Discussion of these competitive effects occupies the first section of the 2010 Guidelines, with market definition contingent upon the identified competitive effects. Indeed, the 2010 Guidelines state, “The Agencies’ analysis need not start with market definition. Some of the analytical tools used by the Agencies to assess competitive effects do not rely on or require market definition” (DOJ and FTC 2010, p. 7).^28^

The 2010 Guidelines also describe competitive effects and market definition as jointly determined: “Evidence of competitive effects can inform market definition, just as market definition can be informative regarding competitive effects” (DOJ and FTC 2010, p. 7). Taken together, these statements suggest a diminished role for market definition by the enforcement authorities, as it is explicitly no longer the required first step to assessing the impact of a given merger.^29^ After 2010, the (sometimes perplexing) central question is: How does one assess the effect on price and output of a potential merger without defining a market?

The 2010 Guidelines provide several categories of evidence that the Agencies consider to be informative of potential adverse competitive effects. These include (1) “historical events, or ‘natural experiments;’” (2) whether there is substantial head-to-head competition between the merging parties; and (3) whether one of the merging firms is a “disruptor” that threatens to displace incumbents (DOJ and FTC 2010, p. 3). Geographic concerns are explicitly addressed in the historical events category, where the Agencies provide as an example that using geographical variation of competition between the merging parties “may be informative of post-merger prices” (DOJ and FTC 2010, p. 3).

Even with the 2010 Guidelines’ de-emphasis of the primary role of market definition in merger enforcement, this version still offers additional details as to how the agencies *would* define markets in those circumstances when it is appropriate or necessary to do so. For the first time since 1982, the HHI thresholds for enforcement policy are relaxed, as well as the doctrinal reliance on market shares (DOJ and FTC 2010, pp. 1, 19). Also, while the 2010 Guidelines preserve the demand substitution focus of market definition as implemented with the HMT (and explained it in greater detail than previous versions), this version does not describe any algorithm to determine the smallest product and geographic markets over which a hypothetical monopolist could exert market power, and no longer indicates the order in which products or geographic areas should be tested. Instead, the discussion of the HMT is centered on a few examples, tendered to help a reader understand the Agencies’ thinking when considering evidence in market definition. For example, when defining product markets the 2010 version states that “[g]roups of products may satisfy the hypothetical monopolist test without including the full range of substitutes from which customers choose” and illustrates this principle with an example.^30^

When defining geographic markets, the 2010 version recognizes the importance of the location of both producers and customers. While the DOJ may have in practice been considering both producer and customer-based approaches in the past when assessing geographic markets (e.g., depending on the potential for price discrimination), the 2010 Guidelines are the first to emphasize these options in detail. The 2010 version states, “The arena of competition affected by the merger may be geographically bounded if geography limits some customers’ willingness or ability to substitute to some products, or some suppliers’ willingness or ability to serve some customers.”

The 2010 Guidelines describe that one must decide whether to start with either the supplier location or the customer location depending on the relevant economic conditions, especially the potential for price discrimination. This language can only elevate the validity to the outside world of geographic market definition exercises that were centered on customer location from the beginning of the analysis. Prior versions of the Guidelines described geographic market definition as an exercise to be conducted around product flows and/or producer locations, with the implications of price discrimination and customer location receiving only passing mention.

As might be obvious by now, the approach to geographic market definition can be quite different, depending on where one starts. In regard to supplier location based markets: “[T]he Agencies normally define geographic markets based on the location of suppliers” if the product is generally being picked up by the customer at the supplier location and there is no “price discrimination based on customer location.” Once the appropriateness of a supplier-based geographic market is determined, this version sheds light upon the ambiguity in implementation that was introduced in earlier versions, by specifying that “sales made by suppliers located in the geographic market are counted, regardless of the location of the customer making the purchase” (DOJ and FTC 2010, p. 14).

In contrast, for customer-based markets: “[I]f price discrimination based on customer location is feasible as is often the case when delivered pricing is commonly used in the industry, then the Agencies may define geographic markets based on the locations of customers” (DOJ and FTC 2010, p. 13). The 2010 Guidelines make clear that in this exercise the geographic markets “encompass the region into which sales are made. Competitors in the market are firms that sell to customers in the specified region. Some suppliers that sell into the relevant market may be located outside the boundaries of the geographic market” (DOJ and FTC 2010, p. 14). To clarify the customer location methodology, the 2010 version provides three examples, which describe examples of when and how a market may be defined around the locations of customers, as well as which sales should be included in the geographic market when defined in this manner.^31^

The presence of price discrimination (or not) always has been an analytical key to determining whether one should begin with supplier location or customer location; these are two very different approaches to geographic market definition. The 2010 Guidelines offer general guidance with regard to how to determine whether price discrimination is in fact present: “For price discrimination to be feasible, two conditions typically must be met: differential pricing and limited arbitrage” (DOJ and FTC 2010, p. 6). In practice, there remains disagreement about how to show whether or not these conditions are present, and hence there can remain disagreement about the appropriate way to approach geographic market definition in a given matter.

When defining geographic markets, the 2010 Guidelines also provide a list of relevant evidence that the Agencies will consider, which varies to some extent from previous versions (DOJ and FTC 2010, p. 14). Six factors exemplify the 2010 Guidelines’ shift towards including analysis for mergers that may facilitate price discrimination. These are:

- How customers have shifted purchases in the past between different geographic locations in response to relative changes in price or other terms and conditions;
- The cost and difficulty of transporting the product (or the cost and difficulty of a customer traveling to a seller’s location), in relation to its price;
- Whether suppliers need a presence near customers to provide service or support;
- Evidence on whether sellers base business decisions on the prospect of customers switching between geographic locations in response to relative changes in price or other competitive variables;
- The costs and delays of switching from suppliers in the candidate geographic market to suppliers outside the candidate geographic market; and
- The influence of downstream competition faced by customers in their output markets (DOJ and FTC 2010, p. 14).

The 2010 Guidelines broadly preserve the definition of market participants as well as elements of the demand-side focus that was in the 1992 and 1997 versions; but this version makes no distinction between “committed” and “uncommitted” entrants. The 2010 version instead distinguishes between firms that could provide rapid supply responses following any post-merger price changes “without incurring significant sunk costs” and firms for which “entry would take place more slowly” (DOJ and FTC 2010, pp. 15–16). The former firms were considered market participants, while the latter firms’ ability to restrict a merger’s adverse effects was measured based on the “timeliness, likelihood, and sufficiency” of entry (DOJ and FTC 2010, p. 28).

While the 2010 Guidelines diminish the primary role of market definition by focusing on competitive effects and remove market definition as a necessary first step in every case, defining markets remains crucial to many cases. In regards to geographic market definition, the 2010 Guidelines adopt a less algorithmic method that spells out two very different approaches to assessing a geographic market and its participants, with the opportunity for—and proof of—price discrimination at its core.

## An Example from the Beer Industry

We illustrate the evolution of geographic market definition over the six Guidelines with a stylized example of the examination of how geographic markets and geographic market power in the 1966 case *United States v. Pabst Brewing Company*^32^ (*Pabst*) might have been defined and assessed differently over three periods: the original 1968 Guidelines; the time covered by the four revisions that occurred from 1982 through 1997; and the most recent 2010 Guidelines.

We choose this case as a teaching example for three reasons: (1) Beer is a relatively homogenous product (especially at that time) that allows us to abstract away from concerns of appropriate product market definition; (2) The beer industry itself has been the subject of repeated merger scrutiny; and (3) *Pabst* was tried before any of the Guidelines were issued. Note that while some facts of this 1966 case are known, we will pose hypothetical facts where appropriate in order to illustrate differences in the emphasis of the Guidelines over time, and we will be explicit when we do so.

The details of *Pabst* are as follows: In 1958, Pabst—the tenth largest brewer in the U.S.—acquired Blatz Brewing Company—the eighteenth largest brewer—to become the nation’s fifth largest brewer with 4.49% of national industry sales. The DOJ challenged the acquisition under Sect. 7 of the Clayton Act: The DOJ argued that the merger’s effect “may be to substantially lessen competition” in the production and sale of beer in three geographic markets: (1) the United States; (2) the three-state area of Wisconsin, Illinois, and Michigan; and (3) Wisconsin. *Pabst* centered on identifying the correct geographic market in order to determine the extent to which the merger would be expected to affect competition.

While the District Court for the Eastern District of Wisconsin dismissed the case based upon an expansive definition of the relevant geographic market for beer as the entire United States, the Supreme Court reversed and remanded the decision based upon a narrow definition of the relevant geographic market limited to Wisconsin as determined by the “the high demand for Pabst beer among Wisconsin residents” (Chirayath 2005, p. 1043).^33^ For ease of illustration, we assume that beer is the proper product market; that Pabst and Blatz sold substantial amounts of beer outside of Wisconsin; and that the majority of beer purchased by Wisconsin residents was made by Pabst and Blatz. These facts are close to the actual facts on the ground in 1958.

The use of the methodology that was popular during the era of the 1968 Guidelines—the Elzinga–Hogarty test—could have offered support to both the Supreme Court’s and the District Court’s decisions in *Pabst*. Developed in part as a response to the *Pabst* ruling, the Elzinga–Hogarty test defined geographic markets with the use of both imports to and exports from a given area. Specifically, the Elzinga–Hogarty test first constructed a candidate market by finding “the minimum area required to account for at least 75% of the appropriate ‘line of commerce’ shipments of that firm.” It then defined two measures to guide geographic market definition—“little in from outside” (LIFO); and “little out from inside” (LOFI)—and compared the computed values of these two measures for the candidate market to threshold values.

In *Pabst*, after collecting shipment data for the merging firms and competitors, an application of the Elzinga–Hogarty test would find that the LIFO and LOFI criterion support different candidate geographic markets. The LIFO criterion indicated that if little of the product consumed in a candidate region was produced outside the candidate region, this “strongly support[ed] the hypothetical geographic market area” (Elzinga and Hogarty 1973, p. 54).

In our *Pabst* hypothetical, the LIFO criterion might be more likely to support a narrow geographic market that is composed solely of Wisconsin, which would provide support to the Supreme Court’s decision. However, the LOFI criterion indicated that the candidate market should be one in which the merging firms make most their sales, with relatively few sales outside “this is an indicator of the propriety of defining that [broader] area as a market” (Elzinga and Hogarty 1973, p. 57). If we assume that Pabst and Blatz both made substantial sales outside of Wisconsin, this would indicate that the market should be expanded to at least include neighboring states—in contrast to the Wisconsin-only market that was defined by the Supreme Court.

Later versions of the Guidelines would emphasize analyses that suggest different levels of support for these various geographic markets (e.g., Wisconsin only or broader). The fact pattern of our example implies that the existence of price discrimination likely would be necessary to support a finding of the narrower proposed geographic market of Wisconsin alone. As described in this paper, while mentioned for the first time in 1982, none of the four versions of the Guidelines from 1982 to 1997 included detailed discussion of price discrimination and its implications for customer-focused geographic market definition. On the other hand, all but one emphasized starting with the producer location as the beginning of a geographic market definition analysis.^34^

If we take the 1992 Guidelines as our example and start with producer location, one would identify the locations of the Pabst and Blatz production facilities, and then consider the customers to whom they make sales. This exercise would include customers both inside and outside Wisconsin.^35^ The question would then be whether Pabst and Blatz could effectively maintain a joint price increase to their *entire* set of customers. Because of the presence of Pabst and Blatz sales in neighboring states, and if we assume the presence of other breweries in those neighboring states, it could likely be shown that other beer would be priced competitively for some meaningful portion of Pabst and Blatz customers outside of Wisconsin. Were this to be true, the HMT would have concluded that a hypothetical monopolist located in Wisconsin, but selling in other states as well, would not have been able to maintain a 5% SSNIP, and therefore the relevant geographic market must be larger than Wisconsin.

In short, as long as Pabst and Blatz sold meaningful amounts of beer outside of Wisconsin, the HMT approach starting with producer location could find a geographic market that was larger than Wisconsin (and potentially even national, as one continued to iterate with larger groups of suppliers and customers), even if the majority of customers in Wisconsin drink Pabst and Blatz beer. On a different note, even if Wisconsin had been deemed a geographic market under the HMT, *Pabst* would likely never have been challenged because the Pabst-Blatz merger in Wisconsin would have failed to meet enforcement thresholds, which were based on post-merger HHI and changes in HHI.^36^

Under the 2010 Guidelines, the *Pabst* case might have avoided market definition altogether, by assessing other types of evidence, such as price effects of prior mergers in the beer industry and an alternative focus on the nature of head-to-head competition between Pabst and Blatz. Were a market definition analysis to have been conducted, it also could have started from a very different place. The 2010 Guidelines emphasize the ability to start either from producer location or customer location depending on the existence of price discrimination. Imagine that one could show that the conditions for price discrimination described in the 2010 Guidelines were in place. For example, differential pricing could exist due to some transportation cost effect as well as different levels of brand loyalty in Wisconsin relative to outside the state. Also arbitrage might be shown to be limited because the resale of beer is constrained for a variety of practical and regulatory reasons.

In this instance, the 2010 Guidelines would give a roadmap to making the claim that were Pabst and Blatz to merge, price discrimination would be possible, even likely, for Wisconsin residents. The 2010 Guidelines would in turn state explicitly that one should move the geographic market definition exercise into a ‘customer location’ approach. If so, this would lead to results that were likely very different than those under the earlier version of the Guidelines. The SSNIP would ask only what the ‘target’ Wisconsin customers would do in the face of a price increase, ignoring the behavior of other customers of Pabst and Blatz. Perhaps, under this fact pattern one would find that, unlike other Pabst and Blatz customers, for Wisconsin customers alone a 5% price increase could be sustained.

Additionally, while in the 1982–1992 period the low HHI of beer in Wisconsin might be given a large amount of weight, in 2010 concentration levels would be only one of several pieces of information used to assess the likelihood that a Pabst-Blatz merger would raise prices. Analysis that is similar to what is described above might reveal that for some subset of customers Pabst and Blatz could do just that—and it would be those customers on whom the Agency would focus.

## Conclusion

While the past is often prologue, in the case of the Merger Guidelines the past (1968 version) represented a significant change in the *administration* of antitrust. For the first time, the Antitrust Division put flesh on the skeleton-like Congressional language of Sect. 7 of the Clayton Act. But the initial version of the Guidelines had a rough and ready congruence with prevailing Supreme Court precedent. Beginning in 1982, and in all versions thereafter, the Guidelines reflect the influence of economics on the *execution* of merger enforcement.

The 1982 Guidelines and their progeny illustrate the imperialism of what Carlyle derisively called the “dismal science.” Changes in the Guidelines represent the Agencies’ response to advances in economic analysis as well as changes in data availability and data analysis. Because of their economic foundation, the Guidelines now carry influence beyond the task of evaluating mergers and acquisitions.

Finally, the Guidelines provide transparency as to how Sect. 7 enforcement is done within the Agencies, which is an effective emendation over the alternatives of opacity and obscurity.

## Footnotes

1 For earlier accounts of the Guidelines’ trajectory, see Werden (1992); see also Werden (2003). All students of market definition have an intellectual debt to Werden.

2 Examples of types of services that may form limited geographic markets include, but are not limited to: labor markets; healthcare markets; and markets for local services. For example, (some) workers may find spatial mobility in response to wage differentials difficult given fixed costs of moving; and (some) healthcare consumers may be unable to pursue higher-quality or lower-price healthcare services in distant geographies due to the cost and time that are associated with travel.

3 For example, knowledge that a distant firm can shift production at low cost to the specified product and ship the produced good to consumers cheaply would suggest a broader geographic market than one defined using only current consumer demand and substitution patterns.

4 *Brown Shoe Co., Inc. v. U.S.*, 370 U.S. 294, 344 (1962).

5 *Brown Shoe Co., Inc. v. U.S.*, 370 U.S. 294, 320 (1962). Emphasis added.

6 “The sales of two distinct products to a particular group of purchasers can also appropriately be grouped into a single market where the two products are interchangeable… In this latter case, however, it may be necessary also to include in that market the sales of one or more other products which are equally interchangeable with the two products” (DOJ 1968a, b, p. 3).

7 See DOJ (1968a, b, p. 3). The degree of vagueness may have been strategic in that it afforded the DOJ more discretion in applying the Guidelines to a particular merger.

8 “While challenging competitively harmful mergers, the Department seeks to avoid unnecessary interference with that larger universe of mergers that are either competitively beneficial or neutral”. Note, the inclusion of a cost-saving efficiencies defense did not mean that the DOJ was not skeptical of efficiency claims by merger proponents. The efficiencies defense had a high bar. The language read: “In the overwhelming majority of cases, the Guidelines will allow firms to achieve available efficiencies through mergers without interference from the Department. Except in extraordinary cases, the Department will not consider a claim of specific efficiencies as a mitigating factor for a merger that would otherwise be challenged”.

9 *United States v. E.I. du Pont de Nemours & Co.*, 351 U.S. 377, 391–92 (1956). Importantly, while the *Cellophane* decision has some facial similarities to the hypothetical monopolist paradigm that was to come, there is a critical difference: The *Cellophane* decision focuses simply on whether the existing seller can increase its existing price. The HMT asks whether a hypothetical monopolist seller in a given candidate market could, or could not, increase its price. To apply the test implied in the *Cellophane* decision is not a substitute for conducting an HMT. White (2008) notes that a focus on the existing seller skews the results of the paradigm because all existing profit-maximizing firms, whether with or without market power, already set their prices to a level that cannot be profitably increased.

10 Adelman wrote: “No matter how the boundaries may be drawn in terms of products or areas, there is a single test: if, within the purported market, prices were appreciably raised or volume curtailed, would supply enter in such amounts as to restore approximately the old price and output? If the answer is “yes,” then there is no market, and the definition must be expanded…. Any other scheme of definition, is not so much “wrong” as meaningless” (Adelman 1959, p. 688).

11 The Elzinga–Hogarty test utilized shipment outflows and inflows to a given geographic to define geographic markets. See Elzinga and Hogarty (1973); see also Elzinga and Swisher (2011).

12 The 1982 Guidelines also included a statement that “[p]rice discrimination requires that sellers be able to identify those buyers [who cannot easily substitute away from the product/geography] and that other buyers be unable profitably to purchase and resell to them”. These are the classical microeconomic conditions required for price discrimination to be sustained. See Varian (1989, p. 599).

13 The HHI is calculated by summing the squares of the individual market shares of all of the firms that are included in the market.

14 The 1982 Guidelines explained that it would not likely challenge any merger in the unconcentrated region, it would not likely challenge a merger with less than a 100 point increase in HHI in the moderately concentrated region, and it would not likely challenge a merger with less than a 50 point increase in HHI in the highly concentrated region.

15 The 1982 Guidelines justified the transition to the HHI from the 1968 Guidelines’ four-firm concentration ratio because the HHI reflects the composition of market shares of the entire market, rather than only the top four firms.

16 Judge Hand stated that a market share of ninety percent “is enough to constitute a monopoly; it is doubtful whether sixty or sixty-four percent would be enough; and certainly thirty-three percent is not.” *U.S. v. Aluminum Co. of America (Alcoa)* 148 F. 2d. 416, 424 (2d Cir. 1945).

17 As a result, courts began to recognize the test’s value and it became a staple of 1980s market definition in cases such as *United States v. Archer-Daniels-Midland Co.*, 866 F.2d. 242 (8th Circuit 1988) and *Coastal Fuels of P.R. Inc. v. Caribbean Petroleum Corp.*, 79 F. 3d. 182 (1st Circuit 1996).

18 What “location” of the merging firms meant in this regard was not made explicit (e.g., a street, a county, a state). We use a state in this example for illustrative purposes.

19 This omission could lead to considerable variation in how the Guidelines were applied in practice, especially outside of the merger context. The 2010 version of the Guidelines was the first to attempt to clarify this with an explicit statement regarding the need to use all customers of a given firm, no matter the customer location.

20 If the existing firms were in Maine and California, rather than Maine and Vermont, with the same customers and sales, the producer location geographic market approach in this example would find a geographic market that is national. But is it really a different marketplace in this instance with regard to the geographic scope of competition? Perhaps to ask the question is to answer it. This is highlighted by the fact that were HHI calculations to be made in this context, they would include sales that were made to all customers throughout the nation, whether the firms were located in Maine and Vermont, or in Maine and California.

21 See DOJ and FTC (1992, pp. 21–24).

22 While the Bertrand model of oligopolies is traditionally cast as studying price competition between firms that produce differentiated but substitutable products, the same model can examine the substitutability of products across different geographies (i.e., spaces). Thus, while the economic theory that highlighted unilateral effects in the 1992 Guidelines had implications for geography, the primary emphasis remained on product differentiation with almost no explicit discussion of its theoretical implications for geography.

23 See DOJ and FTC (1992, pp. 22–24).

24 For example, consider the merger of two firms, A and B, in Chicago that produce the same product as firm C in Charlottesville, which does not currently sell to customers in Chicago, as current prices and transportation costs make selling there unprofitable, and the only other firm in the country that makes the product is very remote. If the merger of firms A and B in Chicago led to a SSNIP that is larger than the difference in transportation costs between the firms, firm C in Charlottesville could and would expand and enter the market in Chicago. In this context, the 1992 Guidelines would consider firm C the next-best substitute, as demand is most likely to be diverted there rather than to the more distant firm, and should be evaluated next as part of the market definition exercise.

25 It is our understanding that notwithstanding that this was the ‘letter of the law’ in the text of the 1992 Guidelines, the practice within the Agencies often was to expand the candidate geographic market if there was a potential new entrant outside that geography that was expected to be able to defeat a SSNIP within it.

26 See DOJ and FTC (1992, pp. 6, 9).

27 For an analysis of this paradigm shift, see Kaplow (2011).

28 For example, the 2010 Guidelines appear to imply that in a proposed merger in which unilateral effects is the concern, one could theoretically conduct a pricing analysis to attempt to predict the impact of merging on the merging firms’ prices, prior to, or in place of, any market definition exercise.

29 At this juncture, the Guidelines deviate from the Court’s prescription that “Determination of the relevant market is a necessary predicate to a finding of a violation of the Clayton Act because the threatened monopoly must be one which will substantially lessen competition ‘within the area of effective competition.’” *U.S. v. E.I. du Pont de Nemours & Co.*, 353 U.S. 593 (1957).

30 See DOJ and FTC (2010, p. 9).

31 See DOJ and FTC (2010, pp. 14–15).

32 *United States v. Pabst Brewing Company*, 384 U.S. 546 (1966).

33 *United States v. Pabst Brewing Company*, 233 F. Supp. 475 (1964).

34 We do not mean to suggest that price discrimination considerations were not raised, or made successfully, at this time in history; instead, we suggest that a straight read of the text of the Guidelines themselves at this time did not emphasize their importance in geographic market definition. The evolution of economic understanding and the fact of parties’ bringing new arguments to merger proceedings is part of what provoked new versions of the Guidelines. The emphasis on price discrimination in the 2010 Guidelines was due to experience over time that the potential for price discrimination was important in assessing the competitive effects of mergers.

35 As described above, the Guidelines at this time did not make explicit which of the firms’ customers were to be included in this analysis. Nevertheless, economic logic would imply, and the 2010 Guidelines made explicit, the need to use all customers of a given firm, no matter the customer location (DOJ and FTC 2010, p. 14).

36 At the time of the *Pabst* case, the pre-merger HHI in the Wisconsin beer industry was 695 and the Pabst-Blatz merger only increased HHI to 981, while the HHI of the national beer industry was 293 with the merger increasing national HHI by only 12 points. See Elzinga and Swisher (2005, p. 262).
